# Microstructure and Properties of CBN Abrasive Blocks with Cu-Sn-Ti Binder Modified by Ceramic Glass Powder

**DOI:** 10.3390/ma19153160

**Published:** 2026-07-23

**Authors:** Huiju Zhang, Duanzhi Duan, Congcong Cao, Chunhui Li, Sumei Zheng

**Affiliations:** 1 The Higher Educational Key Laboratory for Flexible Manufacturing Equipment Integration of Fujian Province, Xiamen Institute of Technology, Xiamen 361000, China; zhanghuiju@xit.edu.cn (H.Z.); lichunhui@xit.edu.cn (C.L.); 2 State Key Laboratory for Manufacturing Systems Engineering, Xi’an Jiaotong University, Xi’an 710049, China; 3 Xiamen Key Laboratory of Intelligent Fishery, Xiamen Ocean Vocational College, Xiamen 361000, China; zhengsumei@xmoc.edu.cn

**Keywords:** Cu-Sn-Ti, CBN abrasives, glass powder, composite binder, self-sharpening, interfacial bonding, wear resistance

## Abstract

Cu-Sn-Ti metallic-bonded CBN (Cubic Boron Nitride) abrasives are widely applied in the precision grinding of superhard materials. However, their high density and high toughness easily lead to poor grain protrusion and inadequate self-sharpening. In this work, ceramic glass powder was incorporated to modify the Cu-Sn-Ti binder, thereby fabricating composite-bonded CBN abrasive blocks with qualified mechanical properties and excellent self-sharpening performance. Flexural strength of abrasive blocks and microhardness of composite binders were measured; microstructure was characterized, phase composition was analyzed by XRD, and tribological tests were carried out between CBN blocks and silicon nitride abrasives. The results indicate that at glass powder contents of 2.94–10.22 wt%, the flexural strength of CBN blocks decreases by 32.5–89.4%, and binder microhardness reduces by 26.5–58.3%. At high brazing temperature, Ti and Cu from Cu-Sn-Ti alloy react with Si and Al in glass powder at the interface to form new phases including Ti_2_O_3_, Ti_5_Si_3_ and Ti(Cu,Al)_2_, which facilitates favorable interfacial bonding among the alloy matrix, glass phase and CBN grains. With increasing glass powder content, the strength decline gradually slows down. The overall wear resistance of abrasive blocks declines. SEM observations on worn CBN blocks and their composite binders reveal the formation of micropores within glass-containing binders, accompanied by a shift in the fracture mode of CBN abrasives upon glass powder addition. Comprehensive experimental analysis indicates that the No.3 sample with 8.33 wt% glass powder possesses the optimal overall performance.

## 1. Introduction

Precision and ultra-precision grinding of superhard materials is the core technology for high-accuracy, high-efficiency and high-quality machining in advanced manufacturing, widely used in aerospace, automotive industry, precision instruments and optical devices [1,2]. Cubic boron nitride (CBN) abrasive tools possess high hardness, good wear resistance, low grinding force and chemical inertness to ferrous metals, and are the optimal choice for precision grinding of difficult-to-machine materials, overcoming the limitation of diamond tools in machining ferrous superhard alloys [3,4]. CBN abrasive tools are divided into metallic-, resin- and ceramic-bonded types [5,6]. Metallic-bonded CBN tools show high bonding strength, good formability, strong grain retention and long service life, and occupy an irreplaceable position in heavy-load, high-efficiency precision grinding [7,8,9].

Among metallic binders, the Cu-Sn-Ti alloy exhibits excellent comprehensive performance [10,11]. The active Ti element realizes chemical wetting and interfacial bonding with CBN grains to enhance bonding strength, while the Cu-Sn matrix provides good toughness, high density and thermal conductivity [10]. It enables the tool to bear complex stress and stably retain abrasive grains during high-speed grinding, ensuring grinding accuracy and service life [12]. Accordingly, Cu-Sn-Ti bonded CBN tools have become a research focus and mainstream application in superhard material precision machining [13,14].

However, the high density, hardness and toughness of Cu-Sn-Ti alloy cause inherent drawbacks [15,16,17]. The binder is hard to wear off in service, leading to insufficient grain protrusion, limited chip space and severe chip clogging, and thus poor self-sharpening [18]. Continuous grinding induces grain passivation, increased grinding force and temperature, easily resulting in workpiece burn, degraded surface quality and reduced machining accuracy, which restricts its application in ultra-precision grinding with high precision and low damage requirements [15,19].

In recent years, extensive research has focused on the modification of copper-based binders to improve the comprehensive grinding performance of CBN abrasive tools [20,21]. Conventional copper binders possess excessively high density and toughness, which suppress binder wear and lead to insufficient abrasive protrusion, inadequate chip accommodation and debris clogging, deteriorating the self-sharpening performance of abrasives [22]. Common modification methods including pore-forming agents, hard ceramic particles and graphite can ameliorate self-sharpening to some extent, yet they either sacrifice mechanical strength for higher porosity or fail to achieve coordinated control over interfacial structure, pore morphology and fracture mode, restricting their application effects [23]. With the merits of low melting point, interfacial reactivity and moderate brittleness, the glass phase is capable of boosting self-sharpening without severe strength loss after being incorporated into Cu-based binders. However, systematic investigations into the influences of glass fraction on interfacial microstructure, pore evolution and fracture mechanism of Cu-Sn-Ti/CBN composites are still lacking.

Although previous studies have confirmed that introducing a glass phase into metal matrices can enhance the comprehensive performance of binders [24], most efforts are limited to single matrix modification or the development of ceramic binders. Systematic and in-depth investigations regarding the precise regulation of interfacial conditions, pore distribution, and fracture behavior in Cu-Sn-Ti bonded CBN abrasive tools via glass phase content are still insufficient. Moreover, the functional mechanism of glass powder during high-temperature fabrication and the interaction characteristics among the glass phase, matrix, and abrasive grains have not been fully clarified, representing a key research gap in this field [25,26]. The core innovation of this study lies in the multi-dimensional regulation of interfacial structure, pore evolution and fracture behavior through glass modification, which fundamentally improves the self-sharpening performance of abrasive tools while ensuring the high grain retention capacity of composite binders [17,27].

CBN abrasive blocks and composite binder specimens with different glass powder contents were fabricated. The effects of glass powder dosage on the mechanical properties, microstructure, interfacial bonding behavior and wear performance of the abrasive blocks were systematically investigated, and the functional mechanism of glass powder during high-temperature vacuum brazing was revealed. This study aims to provide theoretical and experimental support for the development of composite-bonded CBN abrasive tools with high grain retention capability, excellent self-sharpening performance and stable grinding properties [28].

## 2. Materials and Methods

**Figure 1:**
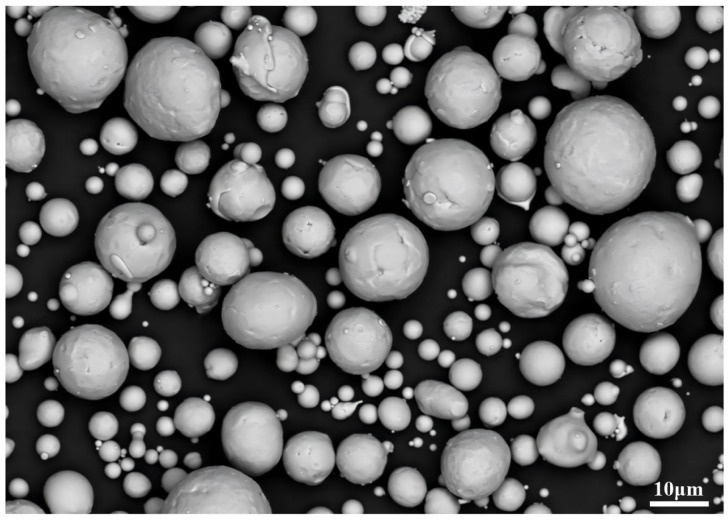
The morphology of Cu-Sn-Ti alloy powder.

**Figure 2:**
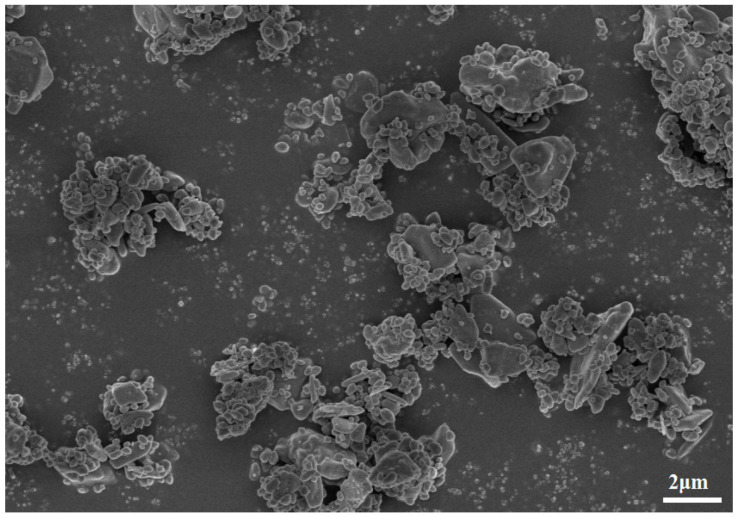
The morphology of glass powder.

Artificial synthetic CBN grains, Cu-Sn-Ti alloy powder (Figure 1), glass powder with a transition temperature of 800 °C (Figure 2) and pure Cu powder were adopted as raw materials in this work. Cubic CBN abrasives with a particle size of 40/45 mesh (Henan Bolairong Superhard Materials Co., Ltd., Zhengzhou, Henan, China) were selected for fabricating CBN abrasive blocks. The mass ratio of the Cu-Sn-Ti alloy powder was set as Cu:Sn:Ti = 65:30:5. The glass powder adopted herein belongs to the SiO_2_-B_2_O_3_-Al_2_O_3_ system, and its chemical composition (mass fraction) is summarized in Table 1, The formulations of the four composite binders used in this study are listed in Table 2.

Raw powders including Cu-Sn-Ti alloy powder, glass powder and Cu powder were weighed with a precision electronic balance according to the mass ratios listed in Table 1, with weighing error controlled within ±0.001 g. 100% pure liquid paraffin was added as milling medium, and the powder mixture was homogenized at ambient temperature for 30 min using an XQM-40 planetary ball mill. The well-blended powders were filled into graphite dies with a cavity dimension of 20 mm × 10 mm × 5 mm and compacted. Subsequent vacuum brazing was carried out in a vacuum furnace at 880 °C under vacuum below 3 × 10^−3^ Pa to fabricate composite binder specimens, with four duplicate samples prepared for each formulation. All specimens were processed by wire electrical discharge machining prior to Vickers hardness measurement, XRD detection, SEM observation and EDS elemental mapping analysis.

**Figure 3:**
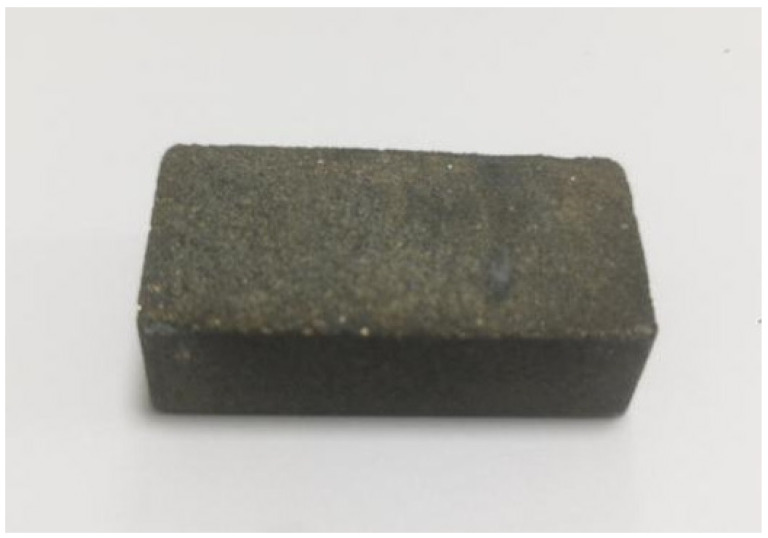
The macroscopic physical morphology of CBN abrasive blocks.

Following identical mixing proportions, procedures and experimental equipment mentioned above, 0.3 ct CBN grits were ultrasonically cleaned in anhydrous ethanol and dried at 80 °C for 1 h to eliminate adsorbed moisture and surface contaminants. The pretreated CBN grains were incorporated into the well-mixed composite binder powders, and the blends were manually stirred for 30 min in a quartz crucible. The resultant mixtures were compacted inside the aforementioned graphite dies and vacuum-brazed under identical furnace parameters to manufacture CBN abrasive blocks, with four replicates prepared for each group. The obtained specimens were subjected to grinding tests, three-point bending measurements and SEM morphological characterization, and the macroscopic appearance of as-prepared CBN blocks is presented in Figure 3.

The microstructure and interfacial characteristics of CBN abrasive blocks were observed via a Hitachi Regulus 8230 scanning electron microscope (SEM), and the elemental distribution was determined by energy dispersive spectroscopy (EDS). X-ray diffraction (XRD, Rigaku SmartLab SE, Tokyo, Japan) was used to identify phase compositions of composite binders, with a step size of 0.05° and a 2θ scanning range of 5–80° [29,30,31,32].

**Figure 4:**
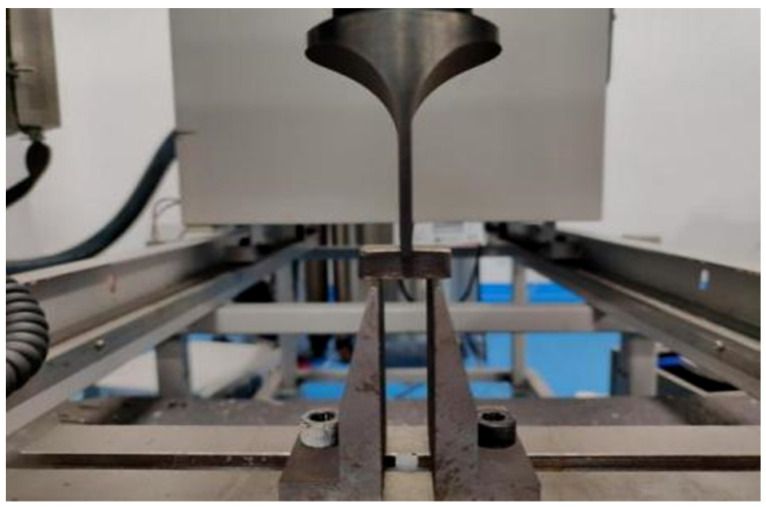
Three-point flexural strength test of CBN abrasive blocks.

Three-point flexural strength tests of CBN blocks were conducted on an INSTRON-5982 universal testing machine (Instron, Norwood, MA, USA). The testing setup is shown in Figure 4. Four CBN abrasive block specimens were prepared for each group, and the reported flexural strength corresponds to the average value of the four replicates. To eliminate the interference of CBN abrasive grains on hardness results, the Vickers microhardness of composite binders was measured using a DHV-1000 Vickers hardness tester (Shanghai Shangcai Testing Machine Co., Ltd., Shanghai, China). The indentation load for hardness testing was set at 1.96 N. Four binder specimens were prepared per group, with five valid indentations measured on each specimen, and the final hardness was averaged from all test data. The influences of glass powder content on the mechanical properties of composite-bonded CBN blocks were analyzed based on the measured three-point flexural strength and Vickers microhardness.

**Figure 5:**
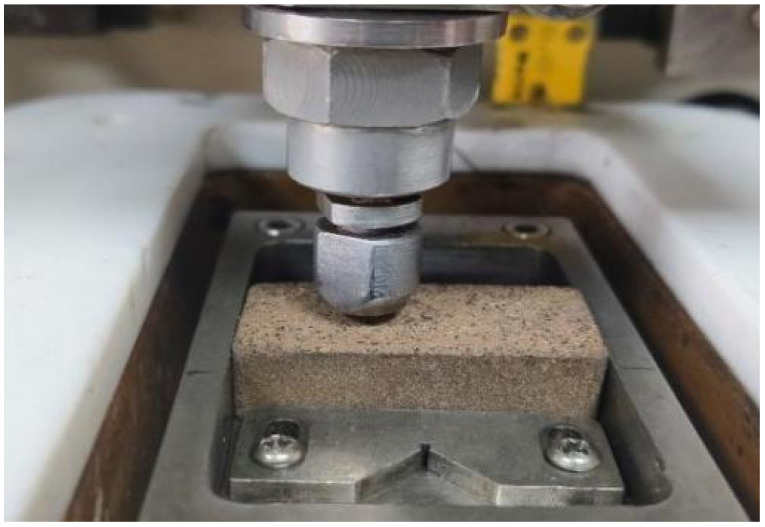
Wear test of CBN abrasive blocks.

Reciprocating friction tests were performed on a CETR UMT3 multifunctional tribometer (CETR, Campbell, CA, USA) using a 6 mm-diameter Si_3_N_4_ ceramic ball as the counterpart. The applied normal load was 40 N, the reciprocating stroke was 5 mm, the reciprocating frequency was 10 Hz, and the total test duration was 30 min. A 10 s preloading stabilization was conducted before testing, and data such as friction coefficient and wear loss were continuously acquired throughout the experiment. The wear test setup is shown in Figure 5. Before and after each test, the CBN blocks were cleaned and dried, and their mass loss (Δm, g) was measured by an analytical balance with an accuracy of ±0.0001 g. Three replicate wear tests were conducted on each of the four CBN abrasive blocks per group to acquire a reliable evaluation of wear performance.

For convenience of description, the CBN abrasive blocks in the subsequent wear tests were named according to the labels of the corresponding composite binders. Specifically, the specimen without glass powder was denoted as the conventional CBN block, and the samples prepared with No.1–No.4 composite binders were labeled as No.1–No.4 CBN blocks, respectively.

## 3. Results and Discussion

### 3.1. Interfacial Characteristics

**Figure 6:**
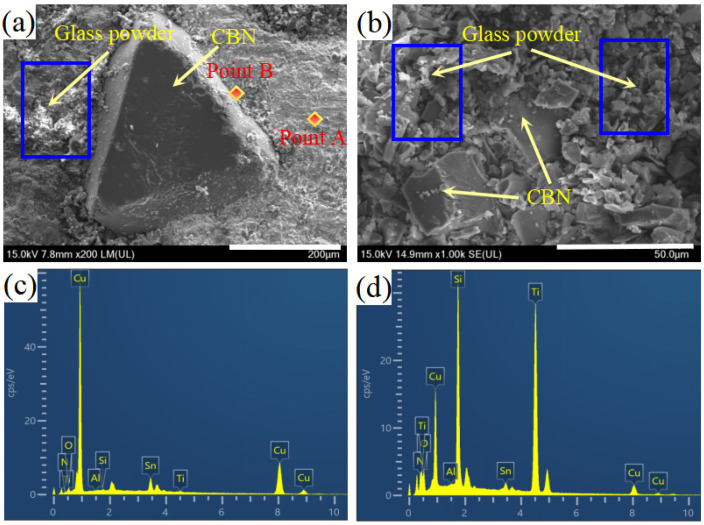
Morphology of No.1 CBN abrasive block: (**a**) SEM morphology; (**b**) SEM morphology; (**c**) element analysis of Point A; (**d**) element analysis of Point B.

The microstructure of CBN abrasive blocks was observed by scanning electron microscopy (SEM). Figure 6 presents the microstructure of the No.1 CBN abrasive block. It can be seen that the CBN grains remain intact without microcracks on the surface. Cu-Sn-Ti alloy powder, Cu powder and glass powder are uniformly distributed around the CBN grains.

A favorable bonding interface is formed between the composite binder and CBN grains, indicating that an appropriate addition of glass powder exerts no adverse effect on the interfacial bonding between Cu-Sn-Ti alloy and CBN abrasive grains. In addition, chemical reactions occur between Cu-Sn-Ti alloy powder and glass powder, generating a well-bonded interfacial structure [33], which will be discussed in detail in Section 3.2.

The Ti content at Point A is 44.08 wt%, more than ten times that of Point B (4.1 wt%). The formation of the Ti-rich zone enhances the chemical reaction tendency between Ti and CBN, promotes the generation of Ti-N and TiB phases, and further improves the interfacial bonding strength. The relevant literature also confirms that Ti-containing active fillers react with CBN during brazing to form Ti-N and TiB phases. Accordingly, a proper dosage of glass powder exhibits no adverse effect on the chemical reaction between CBN grains and the composite binder.

**Figure 7:**
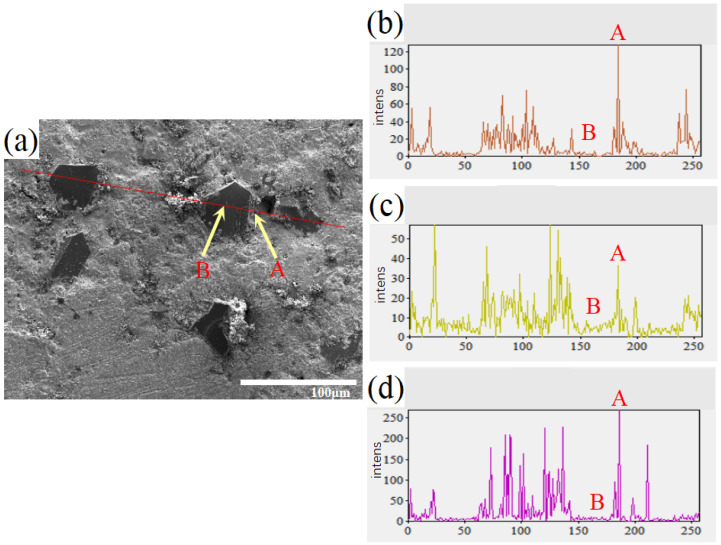
Interfacial characteristics between CBN grain and composite binder of No.2 CBN abrasive block: (**a**) SEM morphology; (**b**) Ti distribution; (**c**) Si distribution; (**d**) Al distribution.

Figure 7 shows the interfacial microstructure of the No.2 CBN abrasive block. As illustrated in Figure 7a, a favorable bonding state is achieved between CBN grains and the composite binder. The linear EDS analysis along the interfacial region is presented in Figure 7b–d. The characteristic peak in Figure 7b indicates that Ti atoms in the composite binder migrate toward the periphery of CBN grains, forming an obvious transition zone.

Figure 7a also reveals the interfacial microstructure between Cu-Sn-Ti alloy powder and glass powder, where a tight bonding interface is formed. EDS analysis of Ti distribution in the interfacial region shows obvious Ti enrichment in the glass powder, indicating that Ti segregates from the molten Cu-Sn-Ti alloy and migrates toward the glass powder [34].

Meanwhile, Si and Al elements diffuse out of the glass powder and migrate to the bonding interface, as shown in Figure 7c,d. This implies the formation of Ti-Si and Ti-Al intermetallic compounds.

**Figure 8:**
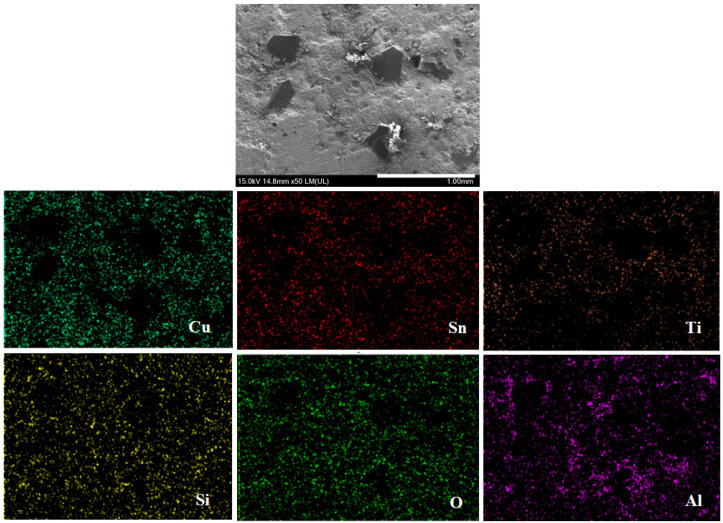
EDS elemental mapping of No.3 CBN abrasive block.

To further verify the above conclusion, surface scanning was performed on the No.3 CBN abrasive block, and the results are shown in Figure 8. Elements including Cu, Sn, Ti, Si, O and Al are uniformly distributed around the CBN grains. This indicates that Ti possesses a high chemical affinity for glass powder at high temperature, facilitating the formation of Ti-Si and Ti-Al intermetallic compounds. The generation of these intermetallic compounds is beneficial to improving the bonding strength between the Cu-Sn-Ti alloy and CBN abrasive grains.

### 3.2. XRD Analysis

**Figure 9:**
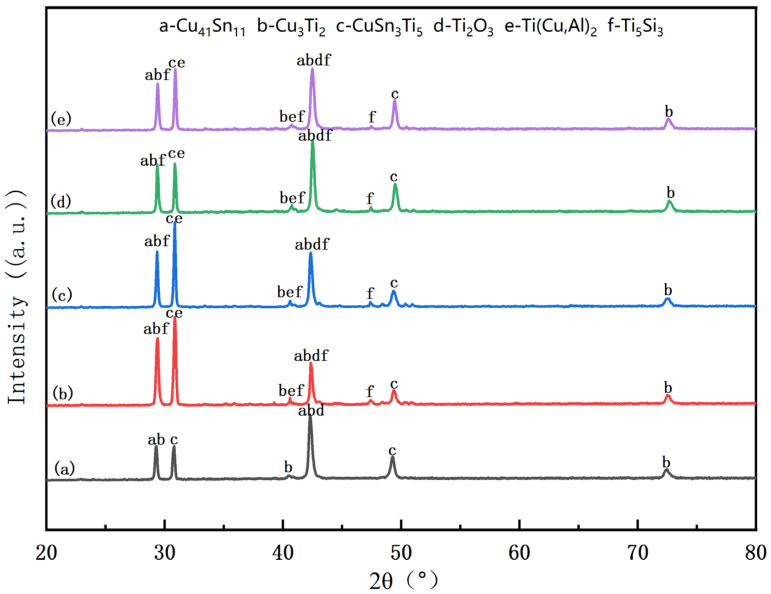
XRD patterns of composite binders: (a) conventional binder; (b–e) No.1 to No.4 composite binders, respectively.

X-ray diffraction (XRD) phase analysis was performed on both conventional and composite binders to clarify phase evolution and interfacial reaction mechanisms during brazing. As displayed in Figure 9a, combined with the previous literature [11,15,17,18,19], the solidified microstructure of glass-free conventional binder is dominated by intermetallic compounds including Cu_41_Sn_11_ (PDF#01-071-0094), CuTi_2_ (PDF#01-071-7900), Cu_4_Ti_3_ (PDF#01-071-7902) and CuSn_3_Ti_5_ (PDF#00-050-1519). These phases are in situ formed via high-temperature reactions between Cu-Sn-Ti alloy powder and pure Cu powder and constitute the primary crystalline constituents of the conventional binder.

Unlike the conventional binder, two new Ti-containing phases, namely Ti_5_Si_3_ (PDF#00-029-1362) and Ti_2_O_3_ (PDF#00-010-0063), are identified in No.1–No.4 glass-modified composite binders via XRD pattern analysis and standard PDF card matching, as presented in Figure 9b–e. This phenomenon originates from the interfacial behavior of active Ti. Under brazing at 880 °C, Ti in Cu-Sn-Ti alloy exhibits strong chemical activity and accumulates at the alloy and glass powder interface through diffusion and segregation, which enables interfacial reactions with SiO_2_ from the glass constituent [35].

According to previous reports [11,25,33], interfacial reactions between molten Ti-bearing alloy and SiO_2_predominantly produce Ti_2_O_3_ and Ti_5_Si_3_, as described by Reaction (1), with the corresponding standard Gibbs free energy function expressed in the Equation of $\Delta G^{0}(\mathrm{kJ}/\mathrm{mol})=\;-1078.97\;-\;0.0406\cdot T$, The calculated standard Gibbs free energy $\Delta G^{0}$ of this reaction is −1125.86 kJ/mol at the brazing temperature of 880 °C (1153 K), demonstrating that the interfacial reaction proceeds spontaneously under the present experimental conditions [36,37].

(1) $${3\mathrm{SiO}}_{2}+9\mathrm{Ti}\overset{1133K}{\to }{\mathrm{Ti}}_{5}{\mathrm{Si}}_{3}+{2\mathrm{Ti}}_{2}O_{3}$$

XRD results further reveal the formation of Ti-Al-containing compounds in Sample Nos. 1–4. Combined with peak matching against the standard PDF card of Al_2_Ti_3_ (PDF#00-029-1362) and the published literature, the emerging phase is confirmed to be Ti(Cu,Al)_2_. This phase originates from high-temperature interfacial reactions among active Ti and Cu from the Cu-Sn-Ti alloy and Al constituent introduced by glass powder. This observation verifies that glass addition facilitates multi-element interfacial reactions. Based on the XRD phase identification and PDF calibration in Figure 9, incorporating glass powder does not degrade the interfacial bonding between CBN grains and binder; instead, stable metallurgical bonding is generated via interfacial chemical reactions to strengthen the interface. Such improvement effectively elevates interfacial bonding strength and load-bearing capacity, laying a favorable phase foundation for robust mechanical retention of CBN abrasives.

### 3.3. Mechanical Properties

**Figure 10:**
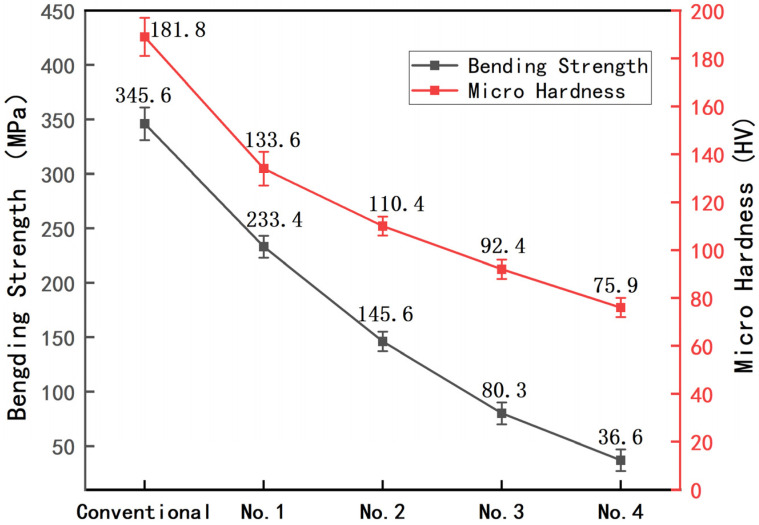
Flexural strength of different CBN abrasive blocks and Vickers hardness of solidified composite binders.

Three-point bending tests were performed on conventional CBN blocks as well as Sample No.1–No.4 CBN blocks. To eliminate the interference of embedded CBN grains on hardness measurement, Vickers microhardness tests were separately carried out on the corresponding binder matrix specimens of all five formulations, and the results are presented in Figure 10. The conventional CBN abrasive block exhibits the highest flexural strength of 345.62 MPa, while the No.4 sample shows the lowest value of only 36.58 MPa. The flexural strength of CBN blocks decreases monotonically with the increase in glass powder mass fraction in the composite binder.

Compared with the conventional CBN block, the flexural strengths of No.1, No.2, No.3 and No.4 specimens decrease by 32.5%, 57.9%, 76.8% and 89.4%, respectively. The declining rate of flexural strength gradually slows down as glass powder content rises, indicating that the deterioration effect of glass powder on the flexural performance follows a trend of significant weakening at first and then gradual moderation. This phenomenon is closely associated with the evolution of interfacial reaction products and the continuity variation in the matrix load-bearing structure.

The conventional binder possesses the highest Vickers microhardness of 181.8 HV, while the No.4 composite binder has the lowest hardness of only 75.90 HV. The microhardness of composite binders decreases continuously with the increasing mass fraction of glass powder.

Compared with the conventional binder, the microhardness of No.1, No.2, No.3 and No.4 composite binders decreases by 26.5%, 39.3%, 49.2% and 58.3%, respectively. In terms of the declining trend, the hardness drop curve gradually becomes gentle from a steep slope with the increase in glass powder content, showing an evolutionary characteristic of rapid initial decline followed by a slow decreasing rate. This reflects that the influence degree of glass powder on binder hardness gradually weakens as its content rises.

### 3.4. Wear Performance

**Figure 11:**
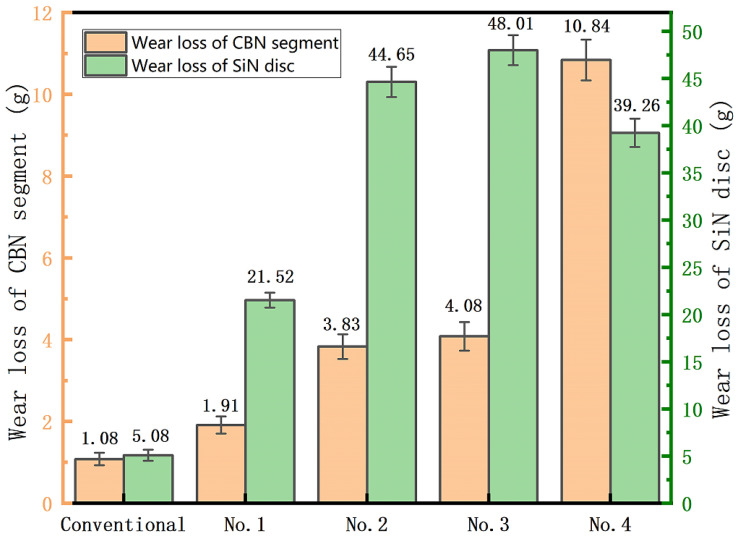
Wear loss of CBN abrasive blocks and Si_3_N_4_ grinding disks after 30 min.

Tribological tests were implemented by sliding conventional CBN blocks and No.1–No.4 CBN blocks with varying glass powder fractions against Si_3_N_4_ counter disks. Figure 11 shows the wear loss of CBN abrasive blocks and Si_3_N_4_ grinding disks after 30 min of grinding. The wear loss of CBN blocks increases gradually with the rise in glass powder content. Compared with the conventional CBN block, the wear losses of No.1, No.2, No.3 and No.4 CBN blocks increased by approximately 76.85%, 162.04%, 287.04% and 903.70%, respectively.

The addition of glass powder reduces the hardness and strength of the composite binder, which aggravates the wear degree and weakens the wear resistance of CBN blocks. This variation is consistent with the mechanical performance trend described in Section 3.3.

The decreased bonding force between the composite binder and CBN grains leads to a higher protrusion height of CBN grains and a weaker holding capacity of the matrix. This improves the self-sharpening ability and sharpness of CBN blocks, thereby enhancing grinding efficiency. The grinding efficiency can be verified by the wear variation in the Si_3_N_4_ disk.

As shown in Figure 11, the wear loss of the Si_3_N_4_ disk presents a non-monotonic trend of first increasing and then decreasing, reaching a peak of 48.01 g at No.3. Relative to the conventional condition, the wear loss of the Si_3_N_4_ disk under No.1, No.2, No.3 and No.4 conditions are about 6.99, 14.50, 15.59 and 12.75 times that of the conventional group, respectively. The wear multiple has peaks at No.3 and then declines slightly at No.4, indicating that the No.3 abrasive block delivers optimal grinding performance.

Grinding ratios calculated from five groups of grinding data in Figure 11 are 4.70, 11.27, 11.66, 11.77 and 3.62, respectively [38]. Analytical results demonstrate that the No.3 specimen achieves a comparatively high grinding ratio. Its moderate and homogeneous microporous structure strikes an optimal balance between lubrication/chip removal and matrix mechanical strength, synchronously acquiring superior cutting efficiency and maximum grinding ratio. Insufficient pores lead to inadequate interfacial lubrication, whereas excessive oversized pores cause structural deterioration; both conditions eventually degrade the overall grinding performance.

To further verify the effect of glass powder addition on the performance of CBN abrasive blocks, the grinding wear rates of the CBN samples in Figure 11 were calculated, and the results are listed in Table 3. Table 3 reveals that compared with the control group, the wear rates of Sample Nos. 1–4 increase by 50%, 225%, 250% and 800%, respectively, with the rising glass powder content, showing a trend from steady growth to accelerated growth. This conclusion is consistent with the hardness and three-point bending strength experimental results presented in Section 3.3, which indicates that the addition of glass powder reduces the wear resistance of CBN abrasive blocks.

The blank control sample and Sample No.1 with a small amount of glass powder exhibit low wear rates and optimal wear resistance. Nevertheless, an excessively low wear rate implies excessively high strength of the binder, which prevents abrasives from timely shedding to expose new cutting edges, deteriorating the self-sharpening property and grinding efficiency. In contrast, Sample No.4 with high glass powder content shows a wear rate of 0.36 mg/min with poor wear resistance; the rapid material loss severely shortens its service life. By comparison, Sample Nos. 2 and 3 possess moderate wear rates of 0.13 mg/min and 0.14 mg/min respectively. Such a moderate wear rate guarantees sufficient service life as well as excellent comprehensive performance. Combined with the grinding ratio data of Sample Nos. 2 and 3, Sample No.3 delivers relatively superior overall grinding performance.

**Figure 12:**
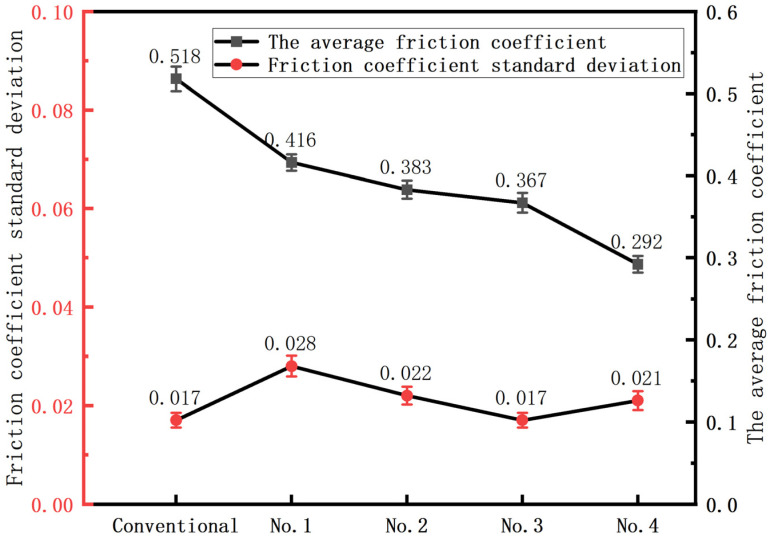
Comparison of average friction coefficient and standard deviation of CBN abrasive blocks.

Figure 12 compares the average friction coefficient and standard deviation of the five groups of CBN abrasive blocks. The conventional sample exhibits high friction resistance and high wear risk, with only acceptable friction stability. Although the traditional metal binder possesses superior mechanical properties, its large friction coefficient results in insufficient self-sharpening performance of CBN abrasive blocks.

All modified samples from No.1 to No.4 achieve friction reduction to varying degrees. Among them, No.3 and No.4 samples reduce the average friction coefficient by 29.1% and 43.6%, respectively, while maintaining the same friction stability as the conventional sample. They demonstrate promising application potential and realize the coordinated optimization of high performance and high reliability without sacrificing friction stability. Combined with the wear experimental results in Figure 11, it is further confirmed that the No.3 CBN abrasive block possesses favorable and stable wear performance.

**Figure 13:**
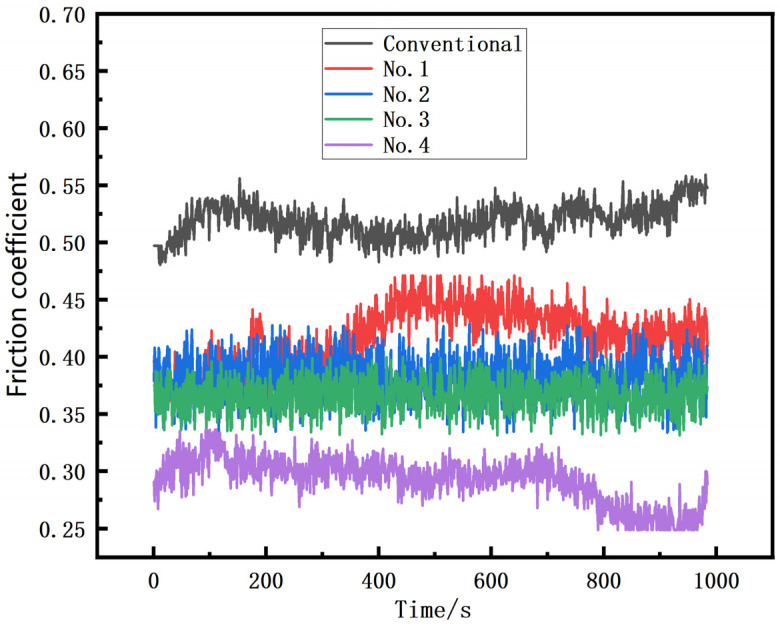
Friction coefficient fluctuation of CBN abrasive blocks.

Figure 13 presents the evolution curves of the friction coefficient over time for the five groups of specimens during a 1000 s friction test, which intuitively reflects the running-in characteristics and steady-state friction behavior of different samples.

The friction levels of the five groups show a distinct hierarchical distribution: conventional (the highest, 0.50–0.55) > No.1 (upper-middle, 0.40–0.46) > No.2 (lower-middle, 0.38–0.40) > No.3 (lower-middle, 0.35–0.38) > No.4 (the lowest, 0.25–0.32). The No.3 specimen achieves a remarkable friction reduction and maintains the most stable friction state throughout the test, without the later-stage performance drift observed in the No.4 sample, thus exhibiting better comprehensive adaptability to actual working conditions.

### 3.5. Mechanism Analysis

**Figure 14:**
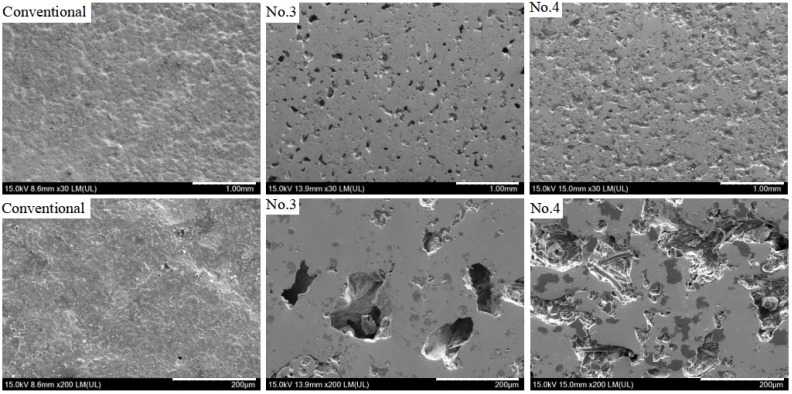
Microstructure of composite binder at 30× and 200× magnifications.

Based on the mechanical properties in Section 3.3 and wear performance in Section 3.4, microstructural characterization was carried out on the conventional, No.3 and No.4 composite binders to reveal the internal mechanism. The characterization results are shown in Figure 14.

The conventional binder without glass powder exhibits a pore-free and highly dense microstructure. The dense structure cannot store lubricant or accommodate wear debris, leading to direct solid–solid contact. Severe adhesion and plowing produce a high and fluctuating friction coefficient, consistent with the previous tribological results.

The No.3 binder is featured with homogeneously scattered micropores without oversized cavities or structural flaws. Its pore size and spatial distribution fall within the optimal range. The uniformly distributed micropores act as abundant micro oil reservoirs to continuously feed lubricant into frictional interfaces during service, while circumventing severe strength degradation and poor load capacity derived from oversized pores. Homogenized stress distribution without localized stress concentration fundamentally restrains the fluctuation of the friction coefficient and endows the composite with outstanding friction stability.

Oversized cavities with uneven pore size are distributed on the surface of the No.4 binder, accompanied by interconnected pores and structural defects in partial regions. Although large pores enhance oil storage and reduce the friction coefficient, they degrade densification and mechanical strength. Under frictional loading, local structural damage and cavity collapse occur easily, inducing friction coefficient drift and poor stability. Additionally, large pores raise local contact stress and aggravate localized wear, weakening long-term service reliability [39].

**Figure 15:**
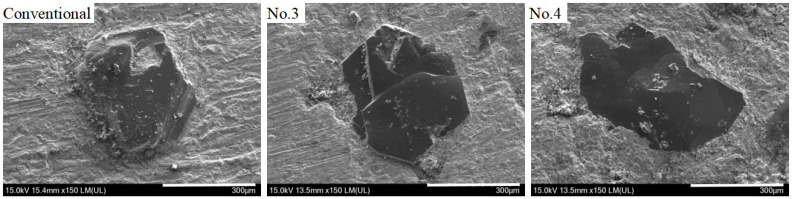
Microstructure of CBN before wear test.

**Figure 16:**
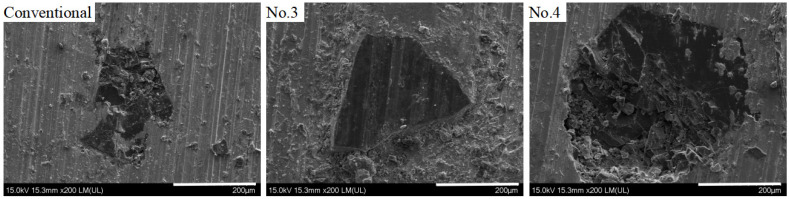
Microstructure of CBN after wear test.

SEM microstructural characterizations were conducted on CBN grains of conventional, No.3 and No.4 specimens before and after grinding tests, with the initial morphology presented in Figure 15 and the post-grinding microstructure shown in Figure 16. Without glass-derived micropores around abrasive grains, the conventional binder features a dense microstructure and superior mechanical strength. Accordingly, binder removal hardly occurs during service, and the dominant failure mode turns into catastrophic bulk fracture inside CBN grains, resulting in inferior self-sharpening performance [39].

Catastrophic bulk fracture is rarely observed for CBN grains in No.3 and No.4 groups; only a small fraction of abrasives suffer from integral or macroscopic cracking, whereas fracture preferentially takes place within the composite binder matrix. This further confirms that the microporous structure induced by glass incorporation weakens the mechanical integrity of the binder. During grinding, the binder is preferentially worn and removed, and cracks initiate inside the binder rather than propagating into CBN grits.

Nevertheless, excessive glass addition in No.4 specimen enlarges pore size and triggers interconnected pores, leading to drastic deterioration in mechanical properties. Despite the enhanced interfacial bonding originating from interfacial chemical reactions between binder and CBN grains, such interfacial reinforcement fails to offset the severe degradation of matrix mechanical performance. Consequently, premature pull-out of CBN abrasives occurs for the No.4 sample, as illustrated in the SEM images of Figure 16.

## 4. Conclusions

(1) SEM characterization confirms that CBN grains in glass-containing specimens remain intact and uniformly distributed without microcracks, verifying that glass powder exerts no adverse effect on the interfacial bonding between CBN grains and the composite binder. EDS results reveal significant diffusion of active Ti from the Cu-Sn-Ti alloy to the binder–CBN interface during high-temperature vacuum brazing. Combined with XRD patterns and standard PDF card indexing, new phases including Ti_5_Si_3_, Ti_2_O_3_, and Ti(Cu,Al)_2_ are detected in glass-modified binders, originating from interfacial reactions between Ti/Cu in the Cu-Sn-Ti alloy and Al/Si from glass powder under brazing conditions. These in situ formed intermetallics strengthen interfacial adhesion, enhance the mechanical retention of CBN grains, and ultimately extend the service life of abrasive blocks.
(2) Compared with the glass-free reference group, composite binders and corresponding CBN blocks with glass powder contents of 2.94 wt%, 5.71 wt%, 8.33 wt%, and 10.22 wt% exhibit degraded mechanical properties: binder microhardness decreases by 26.5%, 39.3%, 49.2%, and 58.3%, while the flexural strength of CBN blocks drops by 32.5%, 57.9%, 76.8%, and 89.4%, respectively. The glass powder-induced mechanical deterioration follows a trend of rapid initial decline followed by gradual slowdown. Notably, a moderate reduction in mechanical performance serves as an effective approach to improving the self-sharpening behavior of CBN grains. As glass powder content modulates self-sharpening performance by tailoring abrasive block mechanical properties, exploring the optimal glass powder addition ratio holds great practical engineering significance.
(3) Reciprocating friction tests against Si_3_N_4_ grinding disks verify the tribological advantages of glass-modified CBN blocks. Among all formulations, the specimen with 8.33 wt% glass powder delivers the optimal grinding performance: the wear loss of its paired Si_3_N_4_ disk reaches 15.59 times that of the conventional CBN sample, with a grinding ratio of 11.77, a wear rate of 0.14 mg/min, and a 29.1% reduction in friction coefficient relative to the unmodified specimen. Post-wear morphological analysis confirms uniform microporous structures in all glass-modified binders. Specifically, the 8.33 wt% glass powder binder features the most homogeneous micropore distribution, and the corresponding specimen fails predominantly via progressive microcrack propagation within the binder matrix rather than transgranular fracture of CBN grains.
(4) Overall, the CBN block with 8.33 wt% glass powder achieves the optimal comprehensive performance. At this doping content, in situ formed interfacial compounds strengthen the bonding between the binder matrix and CBN grains, firmly anchoring the grains and suppressing premature pull-out. Meanwhile, uniformly dispersed micropores moderately reduce binder hardness and strength, inducing progressive matrix micro-fracture during grinding, which enables continuous exposure and renewal of sharp CBN cutting edges and realizes an ideal balance between grain retention and self-sharpening. Furthermore, the microporous structure of the optimized binder accommodates grinding debris and lubricant, effectively suppressing friction fluctuation, improving the grinding ratio, and stabilizing the friction coefficient.

## Figures and Tables

**Table 1:** Chemical compositions and contents in SiO_2_-B_2_O_3_-Al_2_O_3_-based glass powder (wt%).

Chemical Compositions	SiO_2_	B_2_O_3_	Al_2_O_3_	MgO	Others
Contents	55%	25%	10%	5%	5%

**Table 2:** Composition of the composite binders used in this study (wt%).

Process No.	Glass Powder	Cu-Sn-Ti Alloy Powder	Cu Powder
Conventional composite binder	0	60	40
No.1 composite binder	2.94	55.88	41.18
No.2 composite binder	5.71	51.43	42.86
No.3 composite binder	8.33	47.23	44.44
No.4 composite binder	10.22	44.66	45.12

**Table 3:** Mass wear rate *v* of CBN grinding block (mg/min).

CBN Grinding Block Group No.	Conventional	No.1	No.2	No.3	No.4
*v* (mg/min)	0.04	0.06	0.13	0.14	0.36

## Data Availability

The original contributions presented in this study are included in the article. Further inquiries can be directed to the corresponding authors.
